# Forecasting drug utilization and expenditure in a metropolitan health region

**DOI:** 10.1186/1472-6963-10-128

**Published:** 2010-05-17

**Authors:** Björn Wettermark, Marie E Persson, Nils Wilking, Mats Kalin, Seher Korkmaz, Paul Hjemdahl, Brian Godman, Max Petzold, Lars L Gustafsson

**Affiliations:** 1 Department of Drug Management and Informatics, Stockholm County Council, Stockholm Sweden; 2 Division of Clinical Pharmacology, Department of Laboratory Medicine, Karolinska Institutet at Karolinska University Hospital Huddinge, Stockholm Sweden; 3 Department of Oncology and Pathology, Karolinska Insitutet, Stockholm, Sweden; 4 Department of Infectious diseases, Karolinska University Hospital Solna, Stockholm, Sweden; 5 Division of Clinical Pharmacology, Department of Medicine Solna, Karolinska Institutet at Karolinska University Hospital Solna, Stockholm, Sweden; 6 Mario Negri Institute, Milan, Italy; 7 Nordic School of Public Health, Gothenburg, Sweden

## Abstract

**Background:**

New pharmacological therapies are challenging the healthcare systems, and there is an increasing need to assess their therapeutic value in relation to existing alternatives as well as their potential budget impact. Consequently, new models to introduce drugs in healthcare are urgently needed. In the metropolitan health region of Stockholm, Sweden, a model has been developed including early warning (horizon scanning), forecasting of drug utilization and expenditure, critical drug evaluation as well as structured programs for the introduction and follow-up of new drugs. The aim of this paper is to present the forecasting model and the predicted growth in all therapeutic areas in 2010 and 2011.

**Methods:**

Linear regression analysis was applied to aggregate sales data on hospital sales and dispensed drugs in ambulatory care, including both reimbursed expenditure and patient co-payment. The linear regression was applied on each pharmacological group based on four observations 2006-2009, and the crude predictions estimated for the coming two years 2010-2011. The crude predictions were then adjusted for factors likely to increase or decrease future utilization and expenditure, such as patent expiries, new drugs to be launched or new guidelines from national bodies or the regional Drug and Therapeutics Committee. The assessment included a close collaboration with clinical, clinical pharmacological and pharmaceutical experts from the regional Drug and Therapeutics Committee.

**Results:**

The annual increase in total expenditure for prescription and hospital drugs was predicted to be 2.0% in 2010 and 4.0% in 2011. Expenditures will increase in most therapeutic areas, but most predominantly for antineoplastic and immune modulating agents as well as drugs for the nervous system, infectious diseases, and blood and blood-forming organs.

**Conclusions:**

The utilisation and expenditure of drugs is difficult to forecast due to uncertainties about the rate of adoption of new medicines and various ongoing healthcare reforms and activities to improve the quality and efficiency of prescribing. Nevertheless, we believe our model will be valuable as an early warning system to start developing guidance for new drugs including systems to monitor their effectiveness, safety and cost-effectiveness in clinical practice.

## Background

During the last decades of the 20^th ^century, several new and effective drugs have gained widespread use in the treatment of major diseases such as cardiovascular diseases, depression and diabetes mellitus [1]. These drugs markedly decreased mortality, shortened hospital stay and improved the quality of life for large groups of patients. In recent years, science has witnessed breakthroughs in molecular genetics, proteomics and combinational chemistry [2-4]. These advances have triggered the development of biotechnological methods for the design and production of drugs to be used in the diagnosis or therapy of chronic diseases. Consequently many new "biological" drugs have been developed and presently account for 15% of all New Chemical Entities (NCE) or Biological Entities registered annually in US [5]. These drugs are usually considerably more expensive than traditional drugs.

The increasing expenditures for new drugs place considerable pressure on healthcare systems in their efforts to continue to provide comprehensive care [6,7]. As a result, new models for introduction of expensive medicines are urgently needed to avoid prohibitive increases in taxes or health insurance premiums. Such models should include early warning systems (horizon scanning), forecasting of drug utilization and expenditure, critical drug evaluation to help define which patient groups will benefit most from the new medicine, and follow-up to ascertain whether the new drugs are cost-effective in practice.

In the metropolitan health care region of Stockholm, Sweden, a new model to introduce new drugs in healthcare was established in 2007 [8]. The concept is operated through the Regional Drug and Therapeutics Committee (DTC) in Stockholm (LÄKSAK) and includes:

- Early detection (horizon scanning) of drugs to be launched during the coming years

- Forecasting of drug utilization and expenditure

- Critical drug evaluation

- Guidelines for the introduction of medicines, preferably including protocols to assess their value in practice (effect, safety and cost-effectiveness)

- Retrospective quality assessments using observational data

- Communication and involvement of DTC members and professional quality networks, prescribers, patients and other stakeholders at the regional, national and international level

- Continuous monitoring of utilization and expenditure for drugs in hospitals and ambulatory care

- If needed, further educational activities to enhance appropriate prescribing

This paper presents our forecasting model for drug utilization and expenditures, and the predicted growth in different therapeutic areas in the Stockholm region in 2010 and 2011. In addition, we present explanatory factors behind the predicted changes in expenditures. We believe this model will be of interest also for other regions and countries when planning for the future.

## Methods

The forecasting included a prediction of future expenditure in all therapeutic areas based on a linear regression analysis applied to historical sales pattern, subsequently adjusted for factors likely to influence utilization and/or expenditure.

### Linear Regression Analysis

Linear regression analysis was applied to aggregate sales data from the National Corporation of Swedish Pharmacies on hospital sales and dispensed drugs in ambulatory care, including both reimbursed expenditure and patient co-payment. The method of statistical analysis was chosen since the forecasting was based on annual data without seasonal dependencies. We had previously ascertained there were no long term cyclical patterns during few years included. In addition, the resulting residuals from the regression analysis did not show any particular patterns. Consequently, applying time series models without any such patterns would give approximately the same results since the auto-correlations will be estimated to about zero.

Annual expenditures and volumes for all pharmacological groups at the 3rd ATC (Anatomical Therapeutic Chemical Classification) level [9] between 2006 and 2009 were included in the analysis. Expenditure was measured in Swedish Crowns (SEK) (1 Eur = 10.0 SEK, March 2010) and volumes in Defined Daily Doses (DDD) [9]. A linear regression model was applied to each time series of four observations 2006-2009. The crude predictions for the coming two years 2010 and 2011 were based on linear extrapolation. These were then adjusted for factors likely to increase or decrease future utilization and expenditure, such as patent expiries, new drugs to be launched or new guidelines from national bodies or the regional Drug and Therapeutics Committee (Table 1, [10-15]). The individual impact of these factors in each pharmacological group is presented in the results section. No specific adjustments were made for the ageing of the population, population growth and financial incentives for drug prescribing since these changes were already covered by the original trends.

**Table 1:** Factors likely to influence future utilization and expenditure considered in the forecasting model.

Factor	Estimated impact on expenditure	Comment
*Decreasing expenditure*		
Patent expiries and the subsequent introduction of generics	50-90% decrease	In Sweden, since generic substitution was introduced in 2002, reimbursed prices for generics have been decreasing down to 10 to 20% of the price of the original brand within a year after patent expiry [10,11]. Since it may take a year for the prices to decrease by 90%, we estimated expenditure for a drug on an annual basis to be reduced by 50% the first year after patent expiry. We have not applied the same estimates for biosimilars since these are not considered interchangeable and questions still remain about their clinical efficacy, safety, and immunogenicity [12].
	
Changes in prices and reimbursement status	0-20% decrease	All existing drugs are currently being reviewed by the Swedish Dental and Pharmaceutical Benefits Agency (TLV) (value-based pricing for existing drugs) [10]. Individual assessment was performed for each planned reimbursement review since the impact of them has been variable.

*Increasing expenditure*		
Likely new drugs to be launched and new indications for existing drugs	0-x% increase	The potential impact on the healthcare budget was assessed based on estimates of likely/anticipated price for each new product, target patient populations and time for diffusion. Target populations were estimated based on the prevalence and/or incidence of the diseases and conditions or procedures for which each new medicine was likely to be prescribed. Data on the prevalence and incidence were collected from various published and unpublished sources including the Swedish National hospital discharge register, the National prescribed drug register, databases from the County Council, and published scientific studies [13].

*Variable impact*		
New guidelines from national authorities or the regional DTC [11].	+/-5% annual change	Some guidelines were considered to increases in utilization, e.g. National Guidelines for diabetes suggesting stricter targets for HbA1c. Other guidelines were suggested to decrease utilization, e.g. regional guidelines for stricter management of infectious diseases. Overall, guidelines were predicted to have a limited impact during the first two years since prior studies have shown that guidelines are slowly adopted in the healthcare system [14].
	
Introduction of incentives and budgets for drug prescribing along with greater scrutiny of prescribing	+/-0	The regional budgetary model that had been applied for a number of years included voluntary financial incentives for primary care practices linked to the level of adherence to the DTC recommendations and local assessment of prescribing performance in a "prescribing quality report" [15]. A decision had been taken to allocate strict drug budgets for primary care in 2011. However, at the time when the forecasting was performed, it was not clear how it should be constructed. Budgets have also been introduced for ambulatory care prescribing from hospitals. These budgets are, however, only partly allocated and to certain drugs. Consequently, we have not predicted the change in budgeting system to have any impact on the overall trends for 2010-2011.
	
Major structural changes in healthcare provision, organization and reimbursement	0-3% annual increase	A number of structural changes were expected to take place during 2010-2011. A reform increasing patient access to primary healthcare was expected to increase the prescribing of antibiotics, analgesics and antiasthmatics by 3% while changes in access to community pharmacies (state monopoly for pharmacies replaced by new law opening up for private pharmacies) were not expected to influence net expenditure during 2010-2011.

### New Drug therapies or changed indications for old therapies

New therapies were identified through horizon scanning. This is a systematic process to identify new medicines or new indications of existing medicines that are expected to receive marketing authorisation from the Regulatory Authority in the near future and to estimate their potential impact on patient care [16]. Information on drugs likely to be launched during the coming two years was collected from a number of sources. These included published reports and websites from regulatory agencies, the European Commission and the FDA, the UK organizations for horizon scanning (National Horizon Scanning Centre in Birmingham and National Prescribing Centre in Liverpool) and the pharmaceutical industry through pipeline information on their public websites, as well as formal face to face meetings with different pharmaceutical companies.

### Review and modification of forecasting based on expert input

All the information collected was discussed and prioritized in consultation with each of the 23 medical and scientific expert groups belonging to the DTC system in the Stockholm County [10]. These expert groups cover diseases of organ systems e.g. cardiovascular, gastrointestinal and neurological disorders. Each group has members representing all major specialist clinics in the county as well as a general practitioner, a clinical pharmacologist and a pharmacist. All involved experts have to apply a strict policy for declaration of interests including contacts with the pharmaceutical industry [10]. Finally, the forecasting models were scrutinized and modified after input from joint workshops with expert groups and after final input and comments from the main authors, who have extensive clinical pharmacological and/or pharmacotherapeutic knowledge or experience. The final forecasting report for 2010-2011 was published in Swedish in March 2010.

## Results

### Overall forecasting results

**Figure 1:**
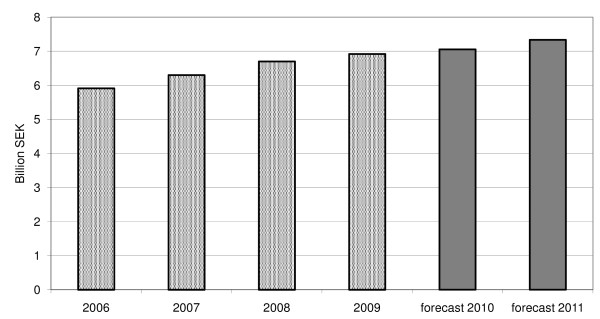
**Historical (2006-2009) and predicted (2010-2011) drug expenditures for the Stockholm County Council**. Hospital sales and dispensed drugs in ambulatory care, including reimbursed expenditure and patient co-payment.

In 2009, the total drug expenditure for the Stockholm County was 6.9 billion SEK, corresponding to 3.490 SEK or €349/inhabitant. This increased by 3.3% compared with 2008 (Figure 1). We predict that the total expenditure, including both ambulatory and hospital sales, will increase by 2.0% in 2010 and by 4.0% in 2011.

**Figure 2:**
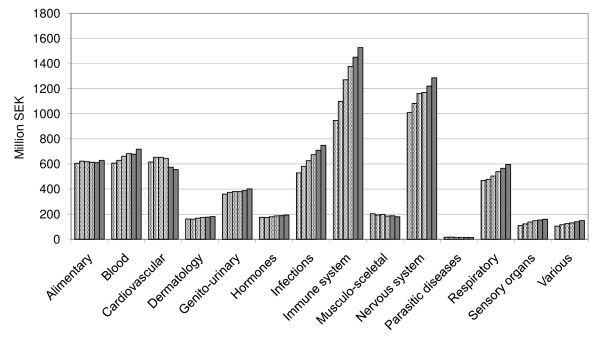
**Historical and predicted drug expenditures 2010 and 2011 (forecast in February 2010) for all pharmacological groups (ATC 1^st ^level) in Stockholm County Council**.

The expenditure is predicted to increase for all major therapeutic areas (Figure 2); most predominantly for antineoplastic and immunomodulating agents (ATC L) as well as drugs for the nervous system (ATC N), infectious diseases (ATC J) and blood-forming organs (ATC B).

In particular, large increases are expected for "biological" drugs, such as TNF-alpha inhibitors and monoclonal antibodies, and small molecules for targeted cancer therapy. We also estimate that expenditure will increase rapidly for some drugs used for common diseases, i.e. drugs prescribed for pain, asthma and diabetes, as well as anticoagulants. This is due to the launch of new medicines, as well as increasing overall volumes without expected savings from new patent expirations. Table 2 contains data on the changes envisaged for all therapeutic groups.

**Table 2:** Historical and predicted drug expenditures for 2010 and 2011 in different therapeutic areas.

ATC	Therapeutic area	Expenditures			Forecast		Change 2010		Change 2011	
		2007	2008	2009	2010	2011	MSEK	(%)	MSEK	(%)
A02	Drugs for acid related disorders	143	124	113	107	104	-5	-5%	-3	-3%
A10	Drugs for diabetes	194	209	214	223	231	9	4%	8	4%
A	Other therapeutic areas	286	286	287	280	293	-7	-2%	13	5%
B01	Anticoagulants	164	180	189	173	202	-16	-9%	29	17%
B02	Coagulation factors	197	212	225	238	249	12	5%	12	5%
B	Other therapeutic areas	267	269	268	267	266	-1	0%	-1	0%
C09C&D	Angiotensin receptor blockers	185	199	204	157	138	-47	-23%	-19	-12%
C10	Lipid lowering agents	145	149	140	131	131	-9	-6%	0	0%
C	Other therapeutic areas	323	305	301	286	287	-14	-5%	1	0%
D	Dermatologicals	161	168	174	177	181	3	2%	4	2%
G	Genito Urinary system	374	382	380	388	401	8	2%	13	3%
H	Hormones	173	179	186	188	192	2	1%	4	2%
J01	Antibiotics	230	243	250	248	250	-2	-1%	2	1%
J05	Antiviral drugs	205	233	270	294	322	24	9%	28	10%
J	Other therapeutic areas	146	151	154	166	175	13	8%	9	5%
L01 & L02	Oncology	521	584	592	591	583	-1	0%	-8	-1%
L04AB	TNF - alpha inhibitors	304	359	420	473	529	53	13%	56	12%
L	Other therapeutic areas	274	328	366	386	415	20	5%	29	7%
M	Musculo-skeletal system	194	198	185	187	180	2	1%	-7	-4%
N02A&B	Analgesics	165	176	189	199	209	10	5%	11	5%
N03	Antiepileptics	122	142	149	157	165	8	5%	7	5%
N05A	Antipsychotics	166	177	175	171	167	-4	-2%	-4	-2%
N06A	Antidepressants	191	187	135	127	127	-8	-6%	0	0%
N	Other therapeutic areas	440	481	522	567	619	44	9%	52	9%
P	Antiparasitic products	17	16	15	15	15	0	-1%	0	-3%
R	Respiratory system	477	504	539	565	596	26	5%	30	5%
S	Sensory organs	123	138	148	154	160	6	4%	6	4%
V	Various	116	125	130	140	148	10	8%	8	6%
A-V	ALL DRUGS	6 304	6 702	6 921	7 056	7 336	135	2%	280	4%

Expenditures are presented in million Swedish Crowns (MSEK) (1 Euro = 10.5 SEK).

### Alimentary tract including antidiabetic agents (ATC-group A)

We expect the expenditures for anti-ulcer drugs to decrease by 5% in 2010 and 3% in 2011 due to patent expiries and decreasing prices for proton pump inhibitors. The impact of lower prices on the overall expenditures will to some extent be counteracted by increasing volumes, although reimbursement restrictions by TLV (Table 1) may limit such an increase [10].

Considerable increases are expected for antidiabetic agents excluding insulin (13% in 2010 and 9% in 2011) due to the increased prevalence of type 2 diabetes [17], new National Guidelines suggesting stricter targets for HbA1c, and the introduction of several new drugs (the GLP-I agonists exenatide and liraglutide, as well as the DPP-4 inhibitors sitagliptin, vildagliptin and saxagliptin) [18]. We anticipate that the safety problems observed for glitazones in patients with ischemic heart disease [19] may contribute to a more rapid introduction of the DPP-IV inhibitors for add-on therapy in patients with type 2 diabetes not controlled on traditional sulphonyl ureas and metformin. On the other hand, there are also anticipated safety problems with these drugs [20]. There is a need for new antidiabetic drugs, but their place in therapy will depend on their long-term safety and effectiveness suggesting a need for the step wise introduction of these NCEs. It is likely that the net increase for all antidiabetic agents will remain at 4% due to the moderation in sales of long-acting insulin as a result of new recommendations in national guidelines and the observational studies demonstrating a potential association between insulin glargin and the development of breast cancer [21].

The ATC group A also includes orphan drugs for patients suffering from rare enzyme deficiencies, e.g. Gaucher's disease. In Europe, orphan drug status can be granted when the prevalence of the disease does not exceed 5 cases per 10 000 inhabitants [22]. Since 2000, when the ad hoc legislation came into force, up to 2007, out of 528 designated orphan indications related to 400 orphan medicinal products (OMP) only 45 (44 drugs) were approved (8.5%) [22]. Access to these drugs varies greatly both between and within countries, mainly because of the high annual cost of treatment (up to € 300 000 per patient) [22]. We predicted the expenditure for orphan drugs within ATC group A to increase by 10% in 2010 and by 9% in 2011.

### Blood and blood forming organs (ATC B)

The expenditure for anticoagulants is likely to decrease by 9% in 2010 and increase by 17% in 2011. The decrease predicted for 2010 is explained by the patent expiry for clopidogrel in November 2009. In March 2010, the price for generic clopidogrel had fallen by 90% compared to the originator price before patent expiry. The subsequent increase in expenditure in 2011 is partly explained by the introduction of dabigatran, rivaroxaban and apixaban [23]. The clinical development of these new anticoagulants is following the well tested strategy of dose-ranging and registration studies for short-term use after major orthopaedic surgery, prior to the development for long-term use in atrial fibrillation. Phase III trials for stroke prevention in patients with atrial fibrillation (AF) are ongoing or recently presented (e.g. RE-LY, Aristotle, Rocket, Borealis and Averroes) [24,25]. Rapid increases in drug expenditure can be anticipated if these drugs will replace warfarin for large numbers of patients [24]. A potential argument for the new more expensive anticoagulant drugs is that increasing drug expenditure may be counteracted by substantial savings in other areas of healthcare if less coagulation control is needed. However, our current opinion is that these drugs also need to be monitored due to the narrow therapeutic margin of anticoagulants, and for the purpose of monitoring compliance. Another factor contributing to the expected increase in antithrombotic drug expenditure is longer treatment periods with clopidogrel in combination with low dose acetylsalisylic acid if the European Guidelines for cardiovascular prevention proposing 12 months treatment [26] are to be adhered to. However, our National Guidelines recommend 3 to 12 months co-treatment and most patients receive shorter co-treatment periods. Lastly, the introduction of prasugrel and tigacrelor may also influence expenditure. Prasugrel has been introduced at a 50% higher price than the original clopidogrel (Plavix), and has recevied reimbursement for treatment of patients with stent thrombosis despite clopidogrel treatment only; this restriction is challenged by the company. However, we do not anticipate that prasugrel will cause major expenses during 2010-2011. Ticagrelor was shown to be superior to clopidogrel in the PLATO study [27], and will presumably be launched in 2011. Its future place in the therapeutic arsenal is so far unclear.

Expenditure for erythropoietin may be reduced due to the introduction of biosimilars but we have not forecasted any major changes in drug expenditure for this group of drugs due to the ongoing uncertainty about differences in biological activity [12]. This may change as more data becomes available about the safety and efficacy of the biosimilars in practice, as well as any significant differences in price between biosimilars and originators in hospital or ambulatory care.

### Cardiovascular drugs (ATC C)

The total expenditure for cardiovascular drugs has remained stable between 2002 and 2009 despite increasing volumes. This is mainly explained by patent expiries and generic availability for some high volume drugs such as simvastatin, amlodipine, enalapril and ramipril during a period when few new drugs were launched [10]. It is likely that expenditures will continue to decrease during the coming two years, mainly due to patient expiries for the angiotensin receptor blockers (ARB's).

As a result of a reimbursement review, ARB's are only reimbursed for patients intolerant to ACE-inhibitors (ACEi) since 2008 [28,29]. This restriction resulted in a 20% decrease in the initiation of ARB while at the same time the number of patients initiated on ACE-inhibitors and calcium channel blockers increased [29]. We believe this change in reimbursement status combined with DTC educational activities and patent expiry of losartan in early 2010 will decrease the expenditure for ARB's by 23% in 2010 and 12% in 2011 (Table 2).

Lipid-lowering agents are the most commonly used drugs in the population after the antiplatelet agents (i.e., mostly low-dose acetylsalicylic acid). In June 2009, the TLV decided on certain reimbursement restrictions, e.g. excluding atorvastatin 10 mg and rosuvastatin 5 mg from the reimbursement scheme [30]. This resulted in a 9% decrease in expenditures. We believe that the expenditures for lipid-lowering drugs will decrease by a further 6% in 2010, but remain stable after that since there is substantial pressure to treat to low cholesterol goals (which will require higher dosages) [31].

Few new cardiovascular drugs will be launched in 2010 and 2011. In 2010, a new antiarrythmic agent, dronedarone, will be introduced as an alternative to amiodarone for patients with atrial fibrillation. The drug has in a large trial (ATHENA) been shown to decrease morbidity and mortality compared to placebo and may, consequently, offer benefits for certain patients [32]. However, the greater tolerability of dronedarone compared to amiodarone is achieved at the expense of lesser efficacy [33], and the target population for dronedarone has not yet been defined.

### Genito-urinary tract and sex hormones (ATC G)

This group is dominated by sex hormones for contraception and hormone therapy for climacteric symptoms. The prescribing of hormone therapy decreased substantially after publication of the randomized studies HERS and WHI in 2002 [34,35]. We predict that the use and consequently expenditure will remain stable during the next couple of years.

The expenditure for drugs for erectile dysfunction is expected to increase by 9% annually due to increasing number of men treated. Further increases in this category of drugs are expected following the launch of the first drug for premature ejaculation (PE), dapoxetin, in 2009. This is because PE is the most common male sexual disorder, estimated to affect up to 30% of men [36]. These "lifestyle" drugs have great potential for increased expenditure since an international comparison showed that Sweden had the most rapid diffusion of sildenafil [37]. However, since then, most drugs for erectile dysfunction have been excluded from reimbursement and there is also more structured diffusion of these drugs through active professional involvement, e.g. in DTC activities [10,38]. This has been factored into the forecast model.

### Infectious diseases (ATC J)

Substantial increases in expenditures are expected for antiviral drugs - 9% and 10%, respectively in 2010 and 2011. We anticipate the expenditure for antiviral drugs will increase due to the continued launch of new drugs for the treatment of HIV and hepatitis B and C. The numbers of patients treated for HIV will increase due to improved survival and ongoing transmission [39]. Furthermore, the emergence of viruses resistant to current drugs is driving the need for new antiretroviral agents [40]. Examples of new drugs include darunavir, maraviroc, enfuvirtide and tifuvirtide. Increasing use of combinations of inhibitors that target different steps of the viral life cycle will also contribute to increasing expenditure.

Increased expenditure is also expected for the treatment of hepatitis. Around 500 new cases of hepatitis C (HCV) are identified in Stockholm each year. This number may increase further with the introduction of retrospective screening for transfusion-transmitted HCV infections [41]. The current treatment of chronic HCV infection consists of a combination of pegylated interferon and ribavirin. New drugs in development include HCV-specific protease inhibitors, polymerase inhibitors, immune modulators and ribavirin analogues [42]. Two of these, thymalfasin and taribavarin, have recently been launched. The management of chronic hepatitis B (HBV) has improved over the last decade with the development of new drugs such as lamivudine, adefovir and dipivoxil, in addition to interferon (IFN)-alpha therapy [43]. Many other new drugs are under development which may contribute to increasing expenditure. These have been factored into the forecast.

The expenditure for antibiotics is predicted to remain stable in 2010-2011. In June 2009, Strama (the Swedish Strategic Programme against Antibiotic Resistance) and the Swedish Institute for Infectious Disease Control (SMI) launched the seventh report on the use of antibiotics and resistance in human medicine in Sweden, Swedres 2008 [44]. The use of antibiotics in Sweden is highest among the elderly and children with prescription rates varying considerably between different regions. The proportion of children aged 0-6 years treated with at least one course of antibiotics in 2008 ranged from 38 per cent in Stockholm county to 25 percent in Västerbotten county, with a national average of 33 percent.

The use of antibiotics both in primary and secondary care seems to be changing in a desirable way, with broad spectrum antibiotics being replaced by narrow spectrum substances. Various types of penicillins have increased and the use of cephalosporins and fluoroquinolones is decreasing. This is in accordance with the guidelines on the reduction of prescription of fluoroquinolones against lower urinary tract infections in women, actively promoted by Strama and the DTCs for many years [45]. There is a certain risk that the decreasing utilization will be counteracted by a recent reform increasing patient access to primary healthcare physicians in Stockholm, so continuous activities are needed. Despite these efforts we expect that expenditure in secondary care will increase in the long run due to a continuous increase in the use of more expensive antibiotics as antimicrobial resistance develops. This will be factored into future predictions.

We expect the expenditure for vaccines to increase by 7% annually. A number of new vaccines with major potential for controlling infectious diseases have just been licensed or are at advanced stages of development. The predicted increases are mainly attributable to the introduction of a new seven- and later 13-valent conjugate pneumococcal vaccine and the use of subsidized vaccines for the prevention of cervical cancer caused by human papilloma virus (HPV) [46,47]. The decision by the Swedish National Board of Health and Welfare that HPV vaccination should be included in the official vaccination program for all girls aged 11-12 years is likely to increase expenditures by 33 million SEK. Completely new or significantly modified vaccines may appear in the future. For the H1N1 influenza, Sweden decided to promote vaccination for its entire population. The vaccine cost for Sweden has been estimated at 1.2 to 1.3 billion SEK and total costs (including administration costs) at around 2.5 billion SEK [48]. This corresponds to a vaccine cost of approximately 0.25 billion SEK for the Stockholm region, more than the total expenditure for analgesics in the region during a year.

### Antineoplastic and immunomodulating agents (ATC L)

**Figure 3:**
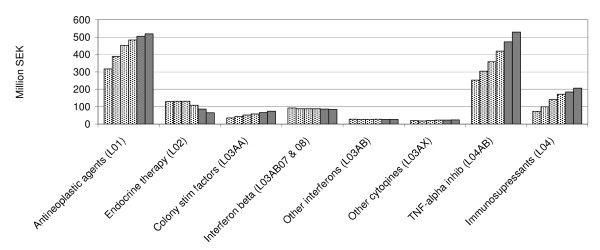
**Historical and predicted drug expenditures 2010 and 2011 for antineoplastic and immunomodulating agents (ATC L) in the Stockholm County Council**.

Increasing expenditure (5% annually) is expected for antineoplastic and immunomodulating agents. The increase is principally confined to immunomodulating agents as we do not expect any increase in the total costs for antineoplastic drugs during 2010-2012 for the reasons outlined below. The increase is mainly due to the widening indications for TNF alpha inhibitors and other drugs already available on the market (Figure 3). Cancer therapy is characterized by multimodal treatment using surgery, radiotherapy and a rapidly increasing number of antitumour agents. Today, most agents are introduced for patients with late stage (metastatic) disease. In many cases such as for example breast cancer, the efficacy in metastatic disease translates into increased cure rates when the new drug is used in earlier stages of the disease in conjunction with surgery [49,50]. Many drugs are under development, and both academic institutions and the pharmaceutical industry are investing in cancer research at levels previously unseen. All of these factors contribute to the predicted increase in expenditure over time, but as discussed below during the time period 2010-2012, this will be counteracted by the patent expiration of several top selling cancer drugs.

Recent advances in the knowledge of the biology of breast cancer have resulted in targeted treatments, such as the monoclonal antibody trastuzumab (targeting HER2-overexpressing cells) [51]. In 2009, trastuzumab was the top selling oncology drug in our region with total sales of 72 million SEK, corresponding to € 7.2 million. However, we predict that the sales will level off during the coming years since the drug has already reached its target population in breast cancer. This will be counterbalanced by trastuzumab recently approved in advanced HER2-overexpressing gastric cancer. However, there are a more limited number of patients.

Colorectal cancer, the third most common malignancy after cancers of the breast and prostate, was treated with surgery alone until the 1980s when the combination of 5-fluorouracil and leucovorin was introduced. During the past ten years, new agents have been introduced and life expectancy has increased from 5 to 20 months in patients with metastatic disease [52]. These improvements have, however, resulted in a dramatic increase in the costs of medical treatment. In recent years, the addition of biological agents like the monoclonal antibodies bevacizumab as well as cetuximab and panitumumab have further improved response rates [53]. None of these drugs has, however, showed activity in the adjuvant setting. Consequently, no major increase in their use in colorectal cancer is expected in the period 2010-2012. The overall use of bevacizumab is expected to increase though mainly due to other indications including advanced breast, lung and renal cancer.

Advances in molecular medicine have also provided insights into the biology of several haematological diseases such as chronic lymphatic leukemia (CLL), chronic myeloid leukemia (CML), multiple myeloma (MM) and non-Hodgkin lymphoma (NHL) [54,55]. This has led to new treatments like the monoclonal antibody rituximab, which has improved survival rates in patients with aggressive NHL and become an important therapeutic option in the treatment of indolent lymphoma [56]. As a result, Rituximab had the second highest expenditure of oncology drugs in the region in 2008 helped also by utilisation in rheumatoid arthritis (RA - below). We predict that sales will continue to grow, mainly due to the widening of indications in haematology (CLL), but also in rheumatoid arthritis (see below). Imatinib has become the backbone of treatment of CML for which several other drugs have also been approved or are in development. Dasatinib and nilotinib are presently approved in second line treatment of CML. If these drugs are approved in first line treatment, this will have a major impact on costs as treatment of CML takes place over many years, and can be considered almost chronic treatment. Bortezomib is indicated in MM for which there are now also several new therapeutic options including lenalidomide [54,55]. Increase in the total drug costs for MM is to be expected.

The rapid increase in expenditure due to new drugs is counteracted by savings due to the introduction of generics for many oncology drugs. The patent recently expired for bikalutamide leading to a rapid price decrease. Over the next two years, patent expiries are expected for several of the best selling oncology drugs including aromatase inhibitors, docetaxel and temozolamide, resulting in the expected moderation in growth in 2010 and 2011. This moderation in growth though is dependant on the rapid uptake of generics at low prices building on previous examples [10]. It is also likely that current resource constraints may increase the need for prioritization of the utilisation of new oncology drugs with only limited improvements in survival in most new cancer drugs compared to current treatments and their high prices. This issue is currently a major focus among health technology assessment units, and in the evaluation of the actual benefit of new cancer drugs [57].

Expenditure has increased rapidly for the treatment of rheumatoid arthritis (RA) due to the introduction of anti-tumor necrosis factor (TNF)-alpha and anti-interleukin (IL)-1 agents (infliximab, adalimumab, etanercept and anakinra). Further increases are expected since the indications have widened and they are increasingly used in patients with inflammatory bowel disease and psoriasis. This considerable widening of indications for TNF-alpha blockers has not though resulted in any price reductions in Sweden. New drugs are also being introduced for the treatment of rheumatoid arthritis patients not responding to conventional treatments, which may also contribute to increasing expenditures. Such drugs include the anti-CD20 monoclonal antibody rituximab, which inhibits B-cell activity, the T-cell activation inhibitor abatacept, and other interleukin inhibitors [58].

### Nervous system (ATC N)

**Figure 4:**
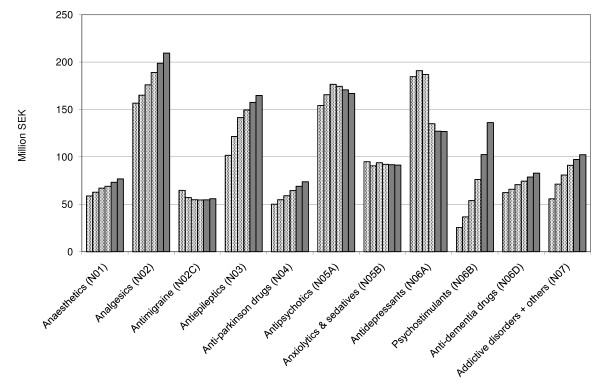
**Historical and predicted drug expenditures 2010 and 2011 for nervous system drugs (ATC N) in the Stockholm County Council**.

We predict the expenditure to decrease for antipsychotics and antidepressants, while it will increase for other CNS acting drugs (Figure 4).

The expenditure on analgesics has increased by 5-13% annually the past years due both to increased volumes and increased prescribing of more expensive products such as tramadol and oxycodon. This development is likely to continue despite efforts from the DTC to moderate their use since they are not considered to provide any advantages over other recommended drugs. New formulations of fentanyl may also contribute to increased expenditures as well as the new fixed combination of naloxone and oxycodon. Some savings will be achieved through patent expiry and the introduction of generics to sumatriptan. This may, however, partly be counteracted by increased utilization of triptans. Other drugs for the treatment of migraine are under development, and it is likely that the first agonist acting on vascular calcitonin-gene related peptide (CGRP) receptors, telcagepant, will be introduced on the market in 2011 [59].

We predict expenditure for antiepileptic drugs to increase by 5% annually, mainly due to increased utilization of lamotrigine and pregabalin for nonepileptic conditions. These include various psychiatric disorders and pain syndromes [60]. Price competition is weak for these drugs since it has been decided nationally that they can not be substituted in pharmacies unlike other generic drugs [10]. Evidence for their benefit in these conditions varies though and further studies are needed to be able to fully determine their place in management. In the USA, the FDA approved pregabalin for treatment of fibromyalgia in June 2007 and the manufacturer filed for the same indication in Europe. However, EMA didn't approve an extension of indication for pregabalin to include the treatment of fibromyalgia [61].

We predict that increase in the expenditure for antipsychotic drugs will moderate over the next two years (Figure 4). This is mainly due to patent expiry and generic competition for risperidone. In 2011, we also expect generics to be introduced for the top-selling drug in the group, olanzapine. Further savings are unlikely though due to the commercial pressures to prescribe the single sourced antipsychotics quetiapine and aripiprazole.

The substantial decrease in expenditure for antidepressants in 2009 is explained by the patent expiry for, and subsequent introduction of generics to venlafaxine (Figure 4). With total sales of 58 million SEK in 2008, venlafaxine accounted for the largest proportion of anti-depressant expenditure in the region. The sales are expected to remain stable over the coming years.

The expenditure for ADHD-medications is expected to increase by 33% annually. The increases are expected both for children and adults. Sweden had earlier a low utilization of these drugs compared to other western European countries, mainly due to strict governmental regulations against the prescription of ADHD medications [62]. Changes in the legislation may partly explain the increasing utilization and also that the disease and the drugs have been heavily discussed in media.

### Sensory organs (ATC S)

The increasing expenditure for ophthalmic preparations has mainly been attributed to the introduction of the vascular endothelial growth factor A (VEGF-A) antagonist ranibizumab to treat neovascular age-related macular degeneration [63]. Studies are ongoing with ranibizumab and other agents for the management of diabetic retinopathy and a widening of the indication is likely to occur in 2011.

## Discussion

Forecasting pharmaceutical utilization and expenditure patterns is a complex undertaking. Factors that drive increases in pharmaceutical expenditures can be divided into increases in price or in volume (i.e. more users and/or longer durations of therapy), and changes in utilization patterns that favour newer, more expensive agents over older, less expensive yet perhaps equally effective alternatives [64]. Part of the increase may also be attributable to the introduction of drugs for diseases that were previously untreatable with existing medications [64].

There are well established methods to predict changes related to these factors, but the traditional models may have difficulties in forecasting the uptake of and expenditures for biological drugs [65,66]. Traditional models may also face problems with reforms and market measures to obtain lower prices for generics and for interchangeable brands in a class if these initiatives are new to the health service. We believe we have addressed both of these issues in our forecasting model for drug utilization and expenditure. Furthermore, the process of including local experts offers the potential to assess early on how regional diffusion rates of new medicines or adoption of guidelines are influenced by local therapeutic traditions and plan for this. Consequently, it is likely that our model may be more accurate to predict future expenditures than macro-forecasting performed at the national level. We would also like to emphasize the importance that the DTC members are involved and balance and question the opinions of the members of the expert groups. A DTC where members have shared evaluation principles and priorities of value of drugs is important as well as transparent declaration of potential conflict of interests for all involved experts according to a guideline [10].

The predicted growth in expenditures for 2010 and 2011 is similar to that found by others. Tuffer et al projected an annual increase of 5,6% in 2010 for prescription drug expenditures in the US [66]. Slightly lower figures were predicted for the US market by Hoffman et al who projected a 0-2% increase in drug expenditures in outpatient settings, a 1-3% increase in expenditures for clinic-administered drugs, and a 1-3% increase in hospital drug expenditures for 2009 [65]. The predicted growth in expenditure is considerable lower than the a 5-7% increase in drug expenditures in outpatient settings, 12-14% increase in clinics, and 4-6% increase in hospitals forecasted by Hoffman and colleagues for 2008 [64]. The moderation is mainly explained by the worldwide economic downturn and growing economic uncertainty [65,66].

The slowdown in growth of ambulatory care drug expenditure in recent years has largely been attributable to the increased use of generics through guidance and financial incentive schemes coupled with market measures to obtain low prices for generics, and there is certain evidence that the era of frequent new "blockbuster" drugs is ending [67-69]. Consequently, the current trend in drug development seems to be switching to personalized and specialized drugs, which emphasizes the need to develop new models to introduce drugs in healthcare.

The development may also be influenced by the financing systems applied in the healthcare system. Financial incentives have been used for many years in primary care in the region [15] and no further substantial changes are expected over the coming years. However, the decision to introduce strict budgets for all hospitals may influence both the overall expenditure and the rate of uptake of new medicines in the future. Financial incentives may moderate the annual increase in drug expenditure and in some cases reduce it. Ethical concerns may be raised as the long term impact of cost containment incentives on the quality of care has not been thoroughly evaluated [70-72].

We acknowledge that there are weaknesses in our model for predicting future drug expenditure. The relative lack of information on launch dates for new products and the selective and delayed publication of results from trials [73] are important problems when trying to estimate the potential value of a new drug and the marketing strategies to come. The pricing of a new drug is also difficult to project unless it is merely another competitor in an established or similar therapeutic class. Early horizon scanning and long term forecasts (more than a couple of years) will thus entail considerable uncertainty. Robust methodologies for horizon scanning [16,74] coupled with the involvement of local experts who keep abreast of developments may, however, provide a reasonable basis for prediction. Other changes in the pharmaceutical market are also difficult to predict, both with regard to timing and effect. For example, estimating patent expiration dates has become a challenge since these dates may change due to litigation, additional patents, exclusivities, and other factors which are difficult to anticipate [75,76]. Another potential limitation is the estimated time for diffusion of new drugs in our model. Diffusion patterns may change due to increased safety concerns with newly approved medications and heightened prescriber sensitivity to the high costs of many new medicines [64]. However, marketing strategies are also changing, and the emergence of personalized medicine with very expensive drugs that are handled by few specialists is creating a new situation that is difficult to evaluate precisely. We believe the utilisation of expert groups in our model helps to address this. Further research is still needed though to determine the current "life cycles" of various therapies as well as factors influencing it.

We are convinced that the model presented here can be developed into more refined models of 'value' based forecasting of drug use in a healthcare region. Value based forecasting can be used to influence treatment patterns through systematic improvements in the selection of patients and drugs for specific pharmacotherapeutic approaches in defined patient populations. Such forecasting may in the future be critical for society to decide priorities and target groups for new expensive treatments or diagnostic procedures compared to the existing ones [77]. Concerns about unethical priorities regarding treatment with expensive drugs could in part be resolved by the use of critical evaluation procedures to elucidate when new drug treatments may be justified. In addition ahead of their launch, determine with key groups which patient populations are likely to most benefit from new drugs and where costs can be controlled by more rational use of generics at low prices and/or established patented alternatives. Solid forecasts are essential in the process of prioritization. A better "prepared" healthcare system will have greater chances of releasing or reallocating resources to help fund valuable new drugs [7,77].

We also believe that it is important to set up registries for monitoring the use of some of the new drugs in specific populations (oncology, rheumatology, MS etc). Such a registry has, for example, been very useful in rheumatology in Sweden [78]. By monitoring the introduction and use of new very costly drugs in special therapeutic areas, a better understanding of their patterns of use and their therapeutic value in practice will be achieved.

Finally, a limitation with our approach is that it only predicts direct expenditure for drugs. New innovative therapies may result in savings in other parts of the healthcare system, such as reduced needs for hospital care or rehabilitation, or reduce societal costs outside of the healthcare system [79,80]. In future models also marginal costs should be considered if other health care costs such as reduced length of stay, and the need for other interventions can be included with some degree of certainty in the estimates. Incorporation of the wider societal perspective also depends on the ability to transfer responsibility and funds between budgets including both health and social care.

## Conclusions

We predicted the annual increase in total expenditure for prescription and hospital drugs to be 2.0% in 2010 and 4.0% in 2011. The utilisation of and expenditure for drugs is difficult to forecast due to uncertainty about the rate of adoption of new medicines and various healthcare reforms and activities to improve the quality and efficiency of prescribing. Nevertheless we believe our model will be valuable as an early warning system to start developing guidance for new drugs including systems to monitor their effectiveness, safety and cost-effectiveness in clinical practice.

## Competing interests

The authors are all involved in the regional Drug and Therapeutics Committee in Stockholm County. No other conflicts of interest.

## Authors' contributions

The model was originally developed by BW, MEP, NW, SK, MK and LLG, and the forecasting for 2010-2011 was performed with all the authors in close collaboration with a large number of experts from the Regional Drug Expert Consortium in Stockholm. In particular, MEP carried out the horizon scanning, BW was responsible for the drug utilization data modelling, MP participated in the statistical analyses, NW, PH and MK participated as pharmacotherapeutic experts in oncology, cardiovascular medicine and infectious diseases, respectively. BG participated as an external advisor coordinating a network with international colleagues. BW was the main responsible coordinating for the manuscript production with active participation from all co-authors. All authors read and approved the final manuscript.

## Pre-publication history

The pre-publication history for this paper can be accessed here:

http://www.biomedcentral.com/1472-6963/10/128/prepub
